# Retraction Note: Pathological dislocation of the hip due to coxotuberculosis in children: a 29-case report

**DOI:** 10.1186/s13018-015-0179-7

**Published:** 2015-03-26

**Authors:** Xin Jiang, Yuan Li, Lijun Liu, MingXin Peng, XueYang Tang, DaoXi Wang, XiaoDong Yang

**Affiliations:** Department of Pediatric Surgery, West China Hospital, Sichuan University, No. 37 Guoxue Road, Chengdu, Sichuan 610041 People’s Republic of China

## Retraction

The Publisher and Editor regretfully retract this article [1] because the peer-review process was inappropriately influenced and compromised. As a result, the scientific integrity of the article cannot be guaranteed. A systematic and detailed investigation suggests that a third party was involved in supplying fabricated details of potential peer reviewers for a large number of manuscripts submitted to different journals. In accordance with recommendations from COPE we have retracted all affected published articles, including this one. It was not possible to determine beyond doubt that the authors of this particular article were aware of any third party attempts to manipulate peer review of their manuscript.

## References

[1] Jiang X, Li Y, Liu L, Peng MX, Tang XY, Wang DX, Yang XD (2014). Pathological dislocation of the hip due to coxotuberculosis in children: a 29-case report. J Orthop Surg Res.

